# Alloantibody among Thalassemia patients receiving multiple blood transfusions at a tertiary care hospital in India

**DOI:** 10.6026/97320630019362

**Published:** 2023-04-30

**Authors:** Sanjay Kumar Thakur, Anil Kumar Sinha, Aarzoo Jahan, Alka Mathur, Dinesh Kumar Negi, Sompal Singh

**Affiliations:** 1 P. G. Department of Zoology, Veer Kunwar Singh University, Ara, Bihar, India 802301; 2 Department of Regional Blood Transfusion Centre, Hindu Rao Hospital and NDMC Medical College, Delhi, India 110007

**Keywords:** Alloantibody, alloimmunization, thalassemia, multiple transfusions

## Abstract

Regular blood transfusion is a lifesaving treatment for thalassemia patients; however, it exposes them to multiple alloantigens. The present study was designed to assess the frequency of alloantibodies in thalassemia patients receiving multiple blood transfusions. Blood samples were tested by Gel card method for ABO, Rh, Direct Antiglobulin Test (DAT), Indirect Antiglobulin Test (IAT), Auto Control (AC) and presence of alloantibody. Alloantibody screening and identification were performed using commercial 3-cell and 11-cell identification panels. Of a total of 66 thalassemia patients, 37 were male and 29 were female, with a mean age of 15.63±5.93 years and a range of 4.0 to 29.0 years. The ABO profiles of thalassemia patients were B-33, A-19, O-11, and AB-3, with 63 Rh-D positives and 3 Rh-D negatives. An average of 533.39±284.95 units were transfused an average of 304±119.65 times. Positive cases for DAT were 29(43.93%), AC was 26(39.39%) and IAT was 4(6.06%). Nine (13.636%) patients had developed alloantibodies, in which anti-K was seen in 5(27.77%), anti-Kp^a^ in 4(22.22%), anti-C in 3(16.66%), anti-C^w^ in 3(16.66%), anti-D in 1(5.55%), anti-Le^a^ in 1(5.55%), anti-Lu^a^ in 1 (5.55%). Alloantibodies were single in 4(44.44%) and multiple in 5(55.55%) patients. The rate of alloimmunization and positivity of DAT, AC, ICT, and splenectomy were significantly associated with higher age, the number of units transfused, and also the number of times of transfusion. Every new thalassemia patient needs extended blood group typing prior to the start of a blood transfusion and antigen-matched blood. For patients with alloantibodies, corresponding antigen-negative blood must be selected for cross-matching.

## Background:

Thalassemia (Greek, *Thalassa*-sea and emia-blood) is the most common heterogeneous group of autosomal recessive, genetic disorder worldwide and is characterized by reduction or absence in synthesis of one or more of the globin chain subunits of Hemoglobin, leading to ineffective erythropoiesis. The type of thalassemia usually carries the name of the affected hemoglobin chain and can be characterised as either alpha, beta or delta thalassemia. Clinically, thalassemia is classified based on their severity into three categories: (1) major-a severe transfusion-dependent anaemia; (2) intermedia-anaemia with splenomegaly and requiring no regular transfusion, and (3) minor-symptomless carrier state. Patients with homozygous beta thalassemia major have an overall deficit of hemoglobin tetramers in their Red Blood Cells (RBCs), decrease in the Mean Corpuscular Volume (MCV) and Mean Corpuscular Hemoglobin (MCH). Clinically, there is pallor, splenomegaly, fever, skeletal abnormalities and failure to thrive. Without stem cell transplantation, the treatment is based on regular blood transfusions (every 3-4 weeks) from early childhood to maintain a mean haemoglobin level of 10-11 g/dL, to improve anaemia, and reduce the skeletal deformities that are associated with excessive erythropoiesis. [1] Although regular blood transfusion is an alternative lifesaving treatment for thalassemia patients, it exposes the patient to multiple alloantigens and introduces living cells into the recipient's body. Due to antigenic differences between donor and recipient blood, blood transfusion leads to the introduction of multiple antigens, such as other red blood cell-specific antigens, human leukocyte antigens (HLA) classes I and II, platelet-specific antigens and granulocyte-specific antigens, into the recipient body. These antigens stimulate the immune system to produce alloantibodies. [2, 3, 4] In transfusion-dependent thalassemia patients, the main cause of the development of red cell alloimmunization is repeated blood transfusion. Blood selection based on ABO and RH-D matching may increase the incidence of alloimmunization in thalassemia. According to Singer *et al*. [5], responsible factors for alloimmunization are complex and the main contributing factors involve at least three elements: (1) the antigenic differences between donor and recipient; (2) the immune status of the blood recipient; and (3) the immunomodulatory effect of alloantigens on the immune system of the blood recipient. [5] A low rate of allo-immunisation is expected when there is homogeneity of red cell antigens between the blood donors and recipients. [5] The rate of allo-immunisation, depending on the blood bank policy and ethnicity, varies worldwide from 4.97% to 37% in different countries and states or provinces worldwide. [6] Therefore, it is of interest to assess the frequency of alloantibody in thalassemia patients receiving multiple blood transfusions to minimize transfusion-associated risk.

## Material and Methods:

A cross-sectional study was performed between the years 2021 and 2022. During the study period, all the thalassemia patients for whom routine transfusion requests were received at the Department of Regional Blood Transfusion Centre, Hindu Rao Hospital, and NDMC Medical College, North Delhi, were included in this study.

## Ethical Considerations:

The study started after the approval of the institutional ethical review committee at Hindu Rao Hospital and NDMC Medical College, North Delhi (F. No.: IEC/NDMC/2021/70, Dated: 19/07/2021). Informed consent was obtained from patients or their legal guardians for all the participants in this study.

## Laboratory Methods:

Blood samples in EDTA and Plan vials were obtained with the patient's blood requisition form. Samples are tested by the Gel card method (DiaClon ABO/D + Reverse Grouping, BIO-RAD, Switzerland) for ABO, Rh, (LISS/Coombs, BIO-RAD, Switzerland), Direct Antiglobulin Test (DAT), Indirect Antiglobulin Test (IAT), Auto Control (AC), and the presence of alloantibodies. Alloantibody screening is performed using a commercial three-cell panel (ID-Diacell I-II-II, Bio-Rad, Switzerland). For the identification of alloantibody, the specificity of all positive screened samples was determined by using a commercial 11-cell identification panel (ID-DiaPanel,Bio-Rad, Switzerland). The 3-cell panel and 11-cell identification panel used in this study were positive for: Rh-hr (D,C,E,c,e,Cw), Kell (K, k, Kp^a^, Kp^b^, Js^a^, Js^b^), Duffy (Fy^a^, Fy^b^), Kidd (Jk^a^, Jk^b^), Lewis (Le^a^, Le^b^), P (P_1_), MNS (M,N,S,s), Luth. (Lua, Lub), Xg (Xga). All the test procedures were performed following the manufacturer's instructions.

Blood Transfusion Protocol: Patients with an alloantibody negative screening result received an ABO and Rh (D) compatible blood transfusion after cross-matching by Gel card method. Alloantibody-positive screened patients received screened antigen-negative RBCs with ABO and Rh (D) compatible blood transfusions after cross-matching by the IAT method. The treating clinician was informed regarding the presence and nature of alloantibodies for further precautions.

## Statistical analysis:

Study data were entered in a Microsoft Excel sheet, and statistical analysis was performed using an open-source Windows statistical software package, Python, version 3.0 (Python, inc., Chicago IL, USA). Analysis was performed for descriptive statistics, frequency distribution, mean and standard deviation. To assess the significant difference of age, number of blood units transfused, time of transfusion among male / female, DAT positive / negative, AC positive / negative, IAT positive / negative, alloantibody positive / negative and splenectomy was assessed using Mann-Whitney U test. To assess the association between other categorical variables, Chi-Square test was used. The P values of less than 0.05 were considered statistically significant.

## Results:

## General details about patients:

A total of 66 thalassemia patients, 37 males and 29 females, were included in this study. The mean age of patients was 15.63 years with a standard deviation of 5.93 years (ranging from 4.0 to 29.0 years). The distribution of ABO blood groups was B-33, A-19, O-11, and AB-3, with Rh-D positive in 63 patients and negative in 3. An average of 533.39±plusmn;284.95 units were transfused to a thalassemia patient. Similarly, a patient was transfused an average of 304±119.65 times. In comparison, a total of 7720 donations were analyzed (healthy donor population), and the distribution of blood groups was as follows: B-2899, A-1791, O-2284, AB-746, with Rh-D positives of 7335 and Rh-D negatives of 385. This difference in the distribution of blood groups between thalassemia patients and healthy donors was statistically significant (X²= 9.06, P= 0.029) the difference in Rh status was not statistically significant (X²= 0.027, P= 0.87).

All patients were tested for DAT, AC, and IAT. DAT was positive in 29 (43.93%) patients and negative in 37 (56.06%) patients; AC was positive in 26 (39.39%) patients and negative in 40 (60.60%) patients; and IAT with ‘O’ positive pool cells was positive in 4 (6.06%) patients and negative in 62 (93.93%) patients (Table 1). Of a total of 66 thalassemia patients, 9 (13.64%) had developed alloantibodies. Of the total of 9 alloantibody positive patients, 5 (27.77%) were positive for anti-K, 4 (22.22%) were positive for anti-Kp^a^, 3 (16.66%) were positive for anti-C, 3 (16.66%) were positive for anti-Cw, 1 (5.55%) was positive for anti-D, 1 (5.55%) was positive for anti-Le^a^, 1(5.55%) was positive for anti-Lu^a^ (a total of 18 antibodies were detected). Out of 9 alloantibody positive patients, 4 (44.44%) have developed a single antibody; 2 (22.22%) have anti-K; 1 (11.11%) has anti-Kpa, 1 (11.11%) has anti-Lea and 5 (55.55%) have developed multiple antibodies: 1 (11.11%) has anti-K with anti-Kp^a^; 1 (11.11%) has anti-D with anti-C^w^; 1 (11.11%) has anti-C with anti-Cw and anti-Kp^a^, 1(11.11%) has anti-C with anti-Cw and anti-K, and 1(11.11%) has anti-C with anti-K, anti-Kp^a^ and anti-Lu^a^. Of a total of 37 male patients, 4 (10.81%) have developed alloantibodies, and of a total of 29 female patients, 5 (17.24%) have developed alloantibodies (Table 2).

## Associations:

There was a statistically significant association between the presence of alloantibodies and AC as well as IAT positivity (P = 0.030 *and* 0.003, respectively, table 3). There was a statistically significant association between DAT positivity and AC positivity (p = 0.000). Similarly, the association between AC positivity and IAT positivity was statistically significant (P = 0.042, Table 3).

A significantly higher (p = 0.000) rate of DAT positivity was observed in the higher age group of patients (mean 18.85 ± 5.66 years) than in the lower age group of patients (13.17 ± 5.82 years). The number of blood units transfused was significantly higher (P = 0.000) in DAT-positive patients (508.98 ± 264.72 units) as compared to DAT negative patients (281.98±235.16 units). Similarly, the number of times the patients were transfused was significantly higher (P = 0.000) in DAT positive patients (373.70 ± 137.54 times) as compared to DAT negative patients (248.74 ± 124.11 times, table 4).

Similar to the results of DAT, AC positive patients had a higher mean age (18.33 ± 5.60 years vs. 13.94 ± 6.31 years, P = 0.001), a higher mean number of blood units transfused (483.64 ± 265.78 units vs. 315.47 ± 257.16 units, P = 0.000) and more average number of times transfusion given (360.23 ± 136.00 times vs. 266.87 ± 137.60 times, P = 0.000, table 4). The IAT positive patients had a higher mean age (P = 0.016), a higher mean number of blood units transfused (P = 0.031) and more average number of times transfusion given (P = 0.018, table 4). The alloantibody positive patients had a higher mean age (P = 0.008), a higher mean number of blood units transfused (P = 0.012), and more average number of times transfusion given (P = 0.011, table 4).

Of a total of 66 patients, 7 had splenectomy; the splenectomized patients had a significantly (P = 0.003) higher age group than patients with a spleen (mean 14.97 ± 6.35 years). The number of blood units transfusion was significantly higher (P = 0.009) in splenectomized patients (613.57 ± 161.02 units) as compared to patients with a spleen (354.21 ± 269.55 units). Similarly, the number of times the patients were transfused was significantly higher (P = 0.011) in splenectomized patients (424.07 ± 107.40 times) as compared to patients with a spleen (289.36 ± 141.14 times, table 4). Splenectomy has no significant association with ABO (P = 0.497), Rh (P = 0.727), Gender (P = 0.642), DCT (P = 0.051), AC (P = 0.154), ICT (P = 0.899), alloantibodies (P = 0.596)

## Discussion:

Alloimmunization is a major blood transfusion problem for thalassemia patients and other patients receiving multiple blood transfusions, as well as for blood transfusion practises. Alloimmunization against RBC antigens occurs due to the introduction of foreign blood cell antigens through blood transfusion, pregnancy, and tissue or organ transplantation. The rate of alloimmunization varies from 4.97% to 37% in studies from different countries. [6]

In our study, we found alloantibodies in 13.64% of the thalassemia patients on repeated blood transfusions. In comparison to the present study, a higher rate of alloimmunization in thalassemia patients (37%) was reported in Taiwan. [7] A study from Arab countries reported that 30% of thalassemia patients had developed red cell alloantibodies. In which anti-K was 72%, anti-E 45.6% and autoantibodies 11%, with and without underlying alloantibodies. [8] A study by Singer *et al*. in the USA has reported that 22% of thalassemia patients had alloantibodies. Among their thalassemic patients, 75% were of Asian descent. [5] A study carried out in Khuzestan province of southwest Iran reported that 18.75% and 12.7%, thalassemia patients had developed alloantibody and autoantibodies respectively, in which 55% was against Rh subgroup system and 33% against Kell system. [9] Hussein *et al*. has reported, alloantibodies in 22.8%, in study from Egypt. They reported 37.4% antibodies against Rh-group (anti-E 14.6%, anti-C 8.9%, anti-D 8.9%, and anti-c 4.9%), followed by anti-Kell (26%), anti-MNS (9.8%), anti-Kidd (8.9%), anti-Duffy (8.1%), anti-Le (5.7%), anti-Lu (2.4%) and anti-P1 (1.6%). They had also reported that, among all Rh-negative patients, Anti-D antibodies developed in 34.5% of cases.[10] Danasoury *et al*. has reported that, in Egyptian thalassemia patients with limited donor exposure program, alloantibodies developed in 19.5% patients. In which Kell and Rh systems was most common.[11] Jansuwan *et al*. had reported in Nan Province of Northern Thailand that, 17.5% thalassemia patients had developed alloantibody.[12]

Reyhaneh et al. have reported, only 30 (1%) patients (Iran, Tehran) developed alloantibodies after screening 3092 occasionally transfused patients. [13] Natukunda et al. (Uganda) have reported that the prevalence of alloantibodies was 6.1% (anti-E, anti-S, anti-D, anti-K and anti-Lea).[14] Spanos *et al*. (Athence, Grees) has reported, after a comparative study of patients who received AB0, rhesus and Kell system-matched blood from their first transfusion, a very low immunisation rate of 3.7%, than those who received only AB0 and Rh-D antigen-matched blood 15.7%. Alloimmunization was 22.6% in thalassemia patients and 36.9% in sickle cell beta-thalassemia patients, and it was significantly associated with the Rhesus (34.0%) and Kell (29.8%) systems. [15] Alloimmunization/autoimmunization in thalassemia patients is reported by Singer *et al*. (25%), Ameen *et al*. (11%), and Dhawan *et al*. 28.2%.[5, 8, 16] In the present study, the frequency of alloimmunization was 13.636% and auto-control was positive in 39.39% of patients. The positivity of auto control was significantly associated with patient age, the number of blood unit's transfusion, times of transfusion and alloimmunization. In our study, patients received AB0 and Rh-D antigen-matched blood, and our centre is situated in Delhi, the capital of India, and most of the patients and donor population are from different states of India. This heterogeneity between the patient and donor populations may be one of the reasons for the slightly higher rate of alloimmunization. Singer *et al*. [5] Hussein *et al*. [10] have reported that the use of leucodepleted blood could have contributed to the low rate of alloimmunization, which is not practised in our centre.

Our results shows, of a total of 66 thalassemia patients, 9 (13.64%) has developed alloantibodies, of a total of 9 alloantibody positive patients, 5 (27.77%) were positive for anti-K, 4 (22.22%) were positive for anti-Kp^a^, 3 (16.66%) were positive for anti-C, 3 (16.66%) were positive for anti-C^w^, 1 (5.55%) was positive for anti-D, 1 (5.55%) was positive for anti-Le^a^, 1 (5.55%) was positive for anti-Lu^a^ (a total of 18 antibodies are detected). Out of 9 alloantibody positive patients, 4 (44.44%) patients has develop single antibody; 2 (22.22%) has anti-K, 1 (11.11%) has anti-Kp^a^, 1 (11.11%) has anti-Le^a^ and 5 (55.55%) has developed multiple antibodies; 1 (11.11%) has anti-K with anti-Kp^a^, 1 (11.11%) has anti-D with anti-C^w^, 1 (11.11%) has anti-C with anti-C^w^ and anti-Kp^a^, 1 (11.11%) has anti-C with anti-C^w^ and anti-K, and 1 (11.11%) has anti-C with anti-K, anti-Kp^a^ and anti-Lu^a^ (Table 2).

Based on various research publications from other states of India, there is heterogeneity in the percentage of allo and autoantibodies as well as type of allo and autoantibodies. Comparing the studies carried out in various regions of India, a higher rate of allo-immunisation of 22.44% was reported in Bankura, West Bengal (93.48% against the Rh phenotype, i.e., c-34.78%, e-26.09%, E+C-19.57%, C-13.06%) and 6.52% against the K antigen. [17] The lower rate of alloimmunization reported in Bhavnagar, Gujarat, India, was 8%. [18] A study from Cuttack, Odisha, India, was 7.5% (anti-E 3.8%, anti-c 1.9%, anti-e 0.9% and anti-D 0.9%).[19] A study from Chandigarh, India was 5.64%, in which Rh system was 52.17% (anti-E 17%, anti-C 13%, anti-D 13%, anti-C^w^ 9%), Kell 35%, Kidd 9%, Xg 4% and 28.2% are autoantibodies.[16] A study from the Jammu region of India found that 8.5% (anti-E 50%, anti D-16.66% anti-K 33.34%) and 1.42% had autoantibodies. [20] A study from Lucknow, India was 8.6% in which 65% were from Rh system. The three most common antibodies detected was anti-E 39.3% followed by anti-K 21.4% and anti-c 10.8% and Autoantibodies in 1.8%.[21] A study from Jaipur, Rajasthan, India, found 7% (anti-D 28.57%, anti-K 28.57%, anti-E 28.57%, and anti-MN 14.3%). They have also reported that, blood group "O" had the highest number (57%) of alloantibodies. [22] A study from Jaipur, Rajasthan, India, has reported alloantibodies in 6.67% [23], which is slightly lower as compared to the present study. The present study results had additional findings that revealed antibodies against more blood groups (anti-Kp^a^, anti-Le^a^ and anti-Lu^a^) as compared to previous studies from India. Compared to the present study, a lower rate of alloimmunization has been reported in other countries. There are low and variable rates of alloantibodies reported in thalassemia patients from Pakistan (6.84%). [4], Shiraz, southern Iran (5.3%) [24], Italy (5.2%) [25], Hong Kong (7.4%). [26], Sulaymaniyah, Iraq (5.8%); [27], Mashhad, Iran (2.87%). [6], Ilam, Iran (10.9%) [28] and Ugandans (6.1%). [14]

Hussein *et al*. [10] have reported that Egyptian males had the highest alloimmunization incidence. In the present study, however, of a total of 29 female patients, 5 (17.24%) have developed alloantibodies, and of a total of 37 male patients, 4 (10.81%) have developed alloantibodies; there is no significant association (p = 0.693) of gender with the frequency of alloimmunization, which is in concordance with the studies carried out by Ameen *et al*. [8]; and Danasoury *et al*. [11].

The present study shows that the development of alloantibody was significantly associated with age (p = 0.008), the number of blood units transfused (p = 0.012), and times of transfusion (p = 0.011), which is in accordance with the studies carried out by Sahu *et al*. [19] and Hussein *et al*. [10] who have reported that patients who receive more units of blood are more likely to show alloimmunization. [10] In a study on patients with different diseases in Uganda, the alloimmunization rate was significantly associated with the number of units of blood transfused as well as the transfusion episodes. [14]

 Hussein *et al*. reported that, splenectomized patients had a higher rate of alloimmunization compared to those who did not have a splenectomy. [10] Jansuwan *et al*. have reported that, alloantibody formation correlates with splenectomy, whereas splenectomy correlates with the number of transfusions. [12] Our results show splenectomy is significantly associated with a higher age group, a higher number of blood units transfused, and a higher number of transfusions. In our study, splenectomy has no significant association with gender, ABO, Rh, alloantibody, DAT, AC, and ICT. The role of splenectomy and the presence of autoantibodies may be associated due to the fact that splenectomy is usually indicated in severe patients requiring multiple transfusions. This confounding effect needs advanced statistical methods and a larger study size.

Though present studies have analyzed a fair number of factors associated with the presence of alloantibodies in thalassemic patients, other factors like ethnicity and genetic makeup can also be analyzed by multinational collaborative study. In our group of thalassemia children, the most common blood group was 'B, followed by 'A', 'O' and AB, whereas in our healthy donor population, the common blood group was 'B' followed by 'O', A, and AB. This difference was statistically significant. In a similar study of patients with thalassemia and hemoglobinopathy from West Bengal, India, Mondal B *et al*. [29] found that blood group 'O' was the most common, followed by blood group B. The difference in blood group profiles of thalassemic patients' needs further study and investigation.

## Conclusion:

The rate of alloimmunization and positivity of DAT, AC, ICT and splenectomy were significantly associated with higher age, the number of units transfused, and the number of times of transfusion. Every new thalassemia patient needs extended blood group typing prior to the start of a blood transfusion and antigen matched blood. For patients with alloantibodies, corresponding antigen-negative blood must be selected for cross-matching.

## Authors' contributions:

The study design was prepared by the authors Sanjay Kumar Thakur, Anil Kumar Sinha, Aarzoo Jahan, Alaka Mthur, Dinesh Kumar Negi and Sompal Singh. The author Sanjay Kumar Thakur performed the literature search, data collection, data analysis and manuscript preparation. All the authors contributed to the data analysis, interpretation, and manuscript preparation. All authors have equally contributed to the preparation and critical review of the final version of the manuscript.

## Funding:

This research did not receive any grant from funding agencies in the public, commercial, not-for-profit sectors.

## Figures and Tables

**Table 1:** Frequency of ABO, Rh, gender and their association with DAT, AC, IAT, Aalloantibody (Allo ab) and Splenectomy of Thalassemia patients.

	**Blood Group**				**Rh**		**Gender**	
	**A (n=19)**	**B (n=33)**	**AB (n=3)**	**O (n=11)**	**+Ve (n=63)**	**-Ve (n=3)**	**M (n=37)**	**F (n=29)**
Male	12	20	2	3	37	0		
Female	7	13	1	8	26	3		
P-value	0.212				0.159			
DAT+Ve	7	14	1	7	26	3	13	16
DAT-Ve	12	19	2	4	37	0	24	13
p-value	0.514				0.159		0.168	
AC+Ve	6	13	1	6	24	2	12	14
AC-Ve	13	20	2	5	39	1	25	15
p-value	0.661				0.7		0.292	
IAT+Ve	0	2	0	2	3	1	2	2
IAT-Ve	19	31	3	9	60	2	35	27
p-value	0.234				0.43		0.788	
Allo ab.+Ve	1	5	0	3	8	1	4	5
Allo ab.-Ve	18	28	3	8	55	2	33	24
p-value	0.333				0.875		0.693	
Splenectomy	2	28	0	11	7	0	5	2
With Spleen	17	5	3	0	56	3	32	27
p-value	0.497				0.727		0.642	

**Table 2:** Frequency of alloantibodies in thalassemia patients

**S.no**	**Age**	**Sex**	**ABO**	**Rh(D)**	**Alloantibody**	
1	18	F	A	+Ve	K	Single antibody 4 (44.44%)
2	15	F	O	+Ve	Kp^a^	
3	23	M	B	+Ve	Le^a^	
4	23	M	B	+Ve	K	
5	20	F	B	-Ve	D, C^w^	Multiple antibody 5 (55.55%)
6	23	F	O	+Ve	C, C^w^, Kp^a^	
7	25	M	O	+Ve	C, K, Kp^a^, Lu^a^	
8	13	M	B	+Ve	K, Kp^a^	
9	21	F	B	+Ve	C, C^w^, K	

**Table 3:** Frequency of DAT, AC, IAT and Alloantibodies and their association with Splenectomy and with each other in thalassemia patients

		**DAT**		**AC**		**IAT**		**Alloantibody**	
		+Ve	-Ve	+Ve	-Ve	+Ve	-Ve	+Ve	-Ve
Splenectomy		6	1	5	2	1	6	1	6
With Spleen		23	36	25	38	3	56	8	51
p-value		0.051		0.154		0.899		0.596	
DAT	+Ve			26	3	4	25	7	22
	-Ve			0	37	0	37	2	35
p-value				0		0.07		0.065	
AC	+Ve					4	22	7	19
	-Ve					0	40	2	38
p-value						0.042		0.03	
IAT	+Ve							3	1
	-Ve							6	56
p-value								0.003	

**Table 4:** Difference between the age group, no. of blood units transfused, Times of transfusion and Gender, DAT, AC, IAT, Alloantibody, Splenectomy, in Thalassemia patients (Mann-Whitney U test).

**Characteristics**	**No. of patients**	**Age**	**No. of blood units transfused**	**Times of transfusion**
Male	37	15.32±6.67	386.77±286.26	302.008±157.12
Female	29	16.11±6.05	375.28±256.25	305.75±126.65
p-value		0.415	0.43	0.456
DAT+Ve	29	18.85±5.66	508.98±264.72	373.70±137.54
DAT-Ve	37	13.17±5.82	281.98±235.16	248.74±124.11
P-value		0	0	0
AC+Ve	26	18.33±5.60	483.64±265.78	360.23±136.005
AC-Ve	40	13.94±6.31	315.47±257.16	266.87±137.60
p-value		0.001	0.001	0.003
ICT+Ve	4	22.00±2.44	619.68±112.64	444.37±77.27
ICT-Ve	62	15.26±6.34	366.37±271.96	294.57±142.34
p-value		0.016	0.031	0.018
Alloab.+Ve	9	20.11±4.044	569.63±215.64	407.25±115.41
Allo ab.-Ve	57	14.97±6.41	352.054±268.95	287.29±141.40
p-value		0.008	0.012	0.011
Splenectomy	7	21.57±2.07	613.57±161.02	424.07±107.40
With Spleen	59	14.97±6.35	354.21±269.55	289.36±141.14
p-value		0.002	0.009	0.011
